# Zinc(II), Palladium(II), and Metal-Free Phthalocyanines Bearing Nipagin-Functionalized Substituents against *Candida auris* and Selected Multidrug-Resistant Microbes

**DOI:** 10.3390/pharmaceutics14081686

**Published:** 2022-08-12

**Authors:** Daniel Ziental, Dariusz T. Mlynarczyk, Emil Kolasinski, Emre Güzel, Jolanta Dlugaszewska, Łukasz Popenda, Stefan Jurga, Tomasz Goslinski, Lukasz Sobotta

**Affiliations:** 1 Chair and Department of Inorganic and Analytical Chemistry, Poznan University of Medical Sciences, Rokietnicka 3, 60-806 Poznan, Poland; 2 Chair and Department of Chemical Technology of Drugs, Poznan University of Medical Sciences, Grunwaldzka 6, 60-780 Poznan, Poland; 3 Department of Engineering Fundamental Sciences, Sakarya University of Applied Sciences, 54050 Sakarya, Turkey; 4 Biomedical Technologies Application and Research Center (BIYOTAM), Sakarya University of Applied Sciences, 54050 Sakarya, Turkey; 5 Chair and Department of Genetics and Pharmaceutical Microbiology, Poznan University of Medical Sciences, Swiecickiego 4, 60-781 Poznan, Poland; 6 NanoBioMedical Centre, Adam Mickiewicz University, Wszechnicy Piastowskiej 3, 61-614 Poznan, Poland

**Keywords:** *Candida auris*, PACT, photosensitizer, phthalocyanine, singlet oxygen

## Abstract

Due to the rapidly increasing problem of antibiotic resistance in recent years, the use of phthalocyanines as photosensitizers with their superior properties in photodynamic antimicrobial therapy (PACT) applications has become important. In this study, magnesium(II) 1,4,8,11,15,18,22,25-octakis(4-[4-butoxycarbonylphenoxy]butyloxy)phthalocyanine was used in the demetalation reaction in trifluoroacetic acid, and subsequently subjected to metalation reaction in dimethylformamide with zinc(II) acetate and bis(benzonitrile)palladium(II) chloride towards zinc(II) and palladium(II) derivatives. Three phthalocyanines, including a demetalated one as well as two metalated, in the core with zinc(II) and palladium(II) were characterized using 1D and 2D NMR spectroscopy and mass spectrometry. In addition, all macrocycles were subjected to absorption and emission studies as well as photostability tests. In a photochemical study, zinc(II) and palladium(II) phthalocyanine complexes appeared to be efficient singlet oxygen generators. There were noted quantum yields of singlet oxygen generation for zinc(II) phthalocyanine derivative in DMF and DMSO at 0.55 and 0.72, whereas for palladium(II) complex at 0.73 and 0.77, respectively. Liposomal formulations of phthalocyanine derivatives were prepared, and their activity was evaluated against a broad spectrum of antibiotic-resistant microorganisms, such as methicillin-resistant *Staphylococcus aureus* (MRSA), *Escherichia coli* (ESBL+), *Candida albicans* resistant to fluconazole, *C. auris*, and against dermatophytes. Phthalocyanine palladium(II) complex showed the highest bactericidal activity against all antibiotic-resistant microorganisms, including reducing *C. auris* growth at 3.54 log.

## 1. Introduction

Due to the rapidly growing problem of antibiotic resistance, photodynamic antimicrobial therapy (PACT) is gaining popularity, especially in the fight against surface infections. We already know today that more and more commonly present microorganisms are becoming resistant to antibiotics, while reports indicate that we live on the threshold of the “post-antibiotic” era. PACT undoubtedly has numerous advantages over traditional antibiotic-based therapy [1,2,3,4]. Finding a photosensitizer (PS) with the best parameters remains a significant challenge. The basic requirement is the high ability to generate singlet oxygen and the stability of the macrocycles at the same time. In the presented article, new liposomal formulations based on very promising compounds from the phthalocyanine (Pc) group were developed. Currently, among the PSs registered so far, those based on porphyrins dominate. However, the development of Pcs has many benefits. First of all, they show poor absorbance in the 400–600 nm range. It is a very valuable feature in PACT, because it significantly reduces the risk of phototoxic reactions in the skin under light at ca. 400 nm due to excitation endogenous porphyrins and mutagenic activity of UV light. The second important issue is that in this range of wavelengths many endogenous substances absorb light, therefore potential PS with absorption between 400 and 600 nm could not be excited efficiently [5]. On the other hand, Pcs are usually poorly soluble in water. This is a major challenge for their medical application [6]. For this reason, there are developing a lot of delivery systems. The best solution for the transportation of Pcs for PDT seems to be liposome vehicles [7].

In PACT, a non-toxic dye (PS) under the influence of light of a specific wavelength undergoes excitation from its ground to an excited state. The molecule returns to its ground state in various ways, including the excitation energy transfer to other molecules and singlet oxygen, which generates reactive oxygen species (ROS). ROS can attack surrounding microorganisms, causing their death due to damage to their membrane or cell wall and organelles [1,2].

Increasing antibiotic resistance accelerated the search for alternative treatment methods for microbial infections. Irresponsible use of antibiotics makes previously used conventional treatments completely ineffective. What is more, the social and economic costs of this problem cannot be ignored. Numerous publications and worldwide reports indicate that infection with antibiotic-resistant bacteria will be one of the most severe medical problems in the coming years [8,9,10,11,12]. Already in 2014, a WHO report indicated that the beginning of the so-called “post-antibiotic era” is not only a possibility but a real threat to humanity [8]. In 2015 alone, over 30,000 people in Europe died due to the inability to treat infections caused by multi-drug-resistant bacteria and because of comorbidities [10]. Antibiotic-resistant bacteria significantly increase the costs of therapy and extend the time of hospitalization. According to the Centers for Disease and Control of Prevention (CDC) estimates, the cost of antibiotic resistance in the United States alone is about 55 billion dollars a year, of which about 35 billion in social costs and 20 billion in healthcare spending [13].

PACT seems to be an excellent adjuvant solution concerning the presented problem. Although its use is limited mainly to surface infections due to limited light penetration, it can be an essential component of other methods of fighting infections that are difficult to treat. PSs used in PACT usually show similar activity against both antibiotic-sensitive and antibiotic-resistant microorganisms [14]. Moreover, the photodynamic action does not cause secondary resistance to other treatments. Due to the extremely short time of ROS activities against microorganisms, these cannot generate specific mechanisms of resistance to PACT [15]. However, searching for the perfect PS candidate is an ongoing challenge [1,2]. Substances with high oxygen generation efficiency, non-toxic, cheap to produce, environmentally friendly, and highly stable in aqueous solutions are still being sought. One of the main areas in developing new PSs is the search for new compounds from the porphyrinoid group [16,17,18,19]. In this way, many new compounds and their combinations with nanocarriers, peptides, and antibodies have been developed [18,20]. In line with this trend, reports indicate the further development of new PSs from the phthalocyanine group and subsequently present their activity against antibiotic-resistant microorganisms and dermatophytes. The use of Pcs seems to be particularly promising in view of the data obtained in recent years. It turns out that PS based on Pcs can be highly effective even against Gram-negative bacteria (such as *E. coli*) [21] and fungi [3] usually more resistant to PACT. The use of nipagins as substituents seems to be very promising. Masilela et al. obtained asymmetric zinc (II) phthalocyanine (ZnPc) with one nipagin substituent which showed good activity against methicillin sensitive *S. aureus* [22]. These results encourage further evaluation of the activity of nipagin-substituted porphyrinoids against antibiotic-resistant microorganisms. In studies conducted so far by Mlynarczyk et al. nipagine-substituted phthalocyanines showed a relatively good safety profile [23]. On the basis of these results, it was decided to try to optimize the structure, achieving the maximum antimicrobial effect while maintaining the minimum dark toxicity. Nipagins, as one of the relatively few substances, exhibit fungistatic activity against a wide spectrum of microorganisms [24,25]. Modification of the PS structure with nipagins suggests that higher activity against fungi will be achieved. So far, fungal infections have been one of the greatest challenges for the developing PACT [3].

The efficacy of presented in this study PSs was tested against Gram-positive bacteria (methicillin-resistant *Staphylococcus aureus*), Gram-negative bacteria (*Escherichia coli* producing extended-spectrum beta-lactamases, ESBL+), fungi (fluconazole-resistant *Candida albicans* and *Candida auris* resistant to most of the currently available antifungals), and dermatophytes (*Trichophyton rubrum* and *Trichophyton mentagrophytes*). Moreover, a potential synergism between selected antimicrobials (meropenem, cephalosporins, gentamicin, ciclopirox, terbinafine, and fluconazole) and various PSs was also analyzed. Particular attention should be paid to the research performed on *C. auris* fungi, which was discovered in 2009 and classified as “super-microbe” [26]. Since approximately 90% of *C. auris* isolates reveal resistance against fluconazole and it is estimated that 4% of infection cases are untreatable [27].

## 2. Materials and Methods

### 2.1. General

All reactions were conducted using *Radleys Heat-On*™ heating system in oven-dried glassware under an argon atmosphere. Solvents and all reagents were obtained from commercial suppliers (Merck, Darmstadt, Germany; Sigma Aldrich, Saint Louis, MO, USA; Chemsolve, Lodz, Poland; Avantor, Gliwice, Poland) and used without further purification, except for dichloromethane, which was distilled before use. All solvents were removed by rotary evaporation at or below 50 °C. A dry flash column chromatography was carried out on Merck silica gel 60, particle size 40–63 µm. Thin-layer chromatography (TLC) was performed on silica gel Merck Kieselgel 60 F_254_ plates visualized with UV (*λ*_max_ 254 or 365 nm). Unless otherwise stated, all mobile phases and solvent mixtures are given in volume to volume (*v*/*v*) ratio. UV-Vis spectra were recorded on a Hitachi UV/VIS U-1900 and Shimadzu U-1900 spectrophotometers. ^1^H and ^13^C NMR spectra were acquired on an Agilent DD2 800 spectrometer at 298 K. Chemical shifts (δ) are reported in parts per million (ppm) and referenced to the residual pyridine-*d*_5_ peak: δ_H_ 8.74, 7.58, 7.22 ppm, δ_C_ 150.35, 135.91, 123.87 ppm). Coupling constants (*J*) are quoted in Hertz (Hz). The abbreviations s, t, and m refer to singlet, triplet, and multiplet. ^1^H and ^13^C resonances were unambiguously assigned based on ^1^H-^1^H COSY, ^1^H-^13^C HSQC, and ^1^H-^13^C HMBC experiments. Mass spectra (MS ES, HRMS ES, MALDI TOF) were carried out by the Wielkopolska Center for Advanced Technologies at Adam Mickiewicz University in Poznan and the Chair and Department of Inorganic and Analytical Chemistry at the Poznan University of Medical Sciences. Magnesium(II) 1,4,8,11,15,18,22,25-octakis(4-[4-butoxycarbonylphenoxy]butyloxy)phthalocyanine (**1**) was prepared according to the previously reported methodology [23].

### 2.2. Synthesis of Pc Derivatives

#### 2.2.1. 1,4,8,11,15,18,22,25-Octakis(4-[4-butoxycarbonylphenoxy]butyloxy)phthal-ocyanine (**2**)

A demetalation reaction was applied from a literature protocol [28]. **1** (200 mg, 0.075 mmol) was dispersed in 5 mL of trifluoroacetic acid. The reaction mixture was stirred at room temperature for 30 min and protected from light. After that, the reaction mixture was poured on ice and water mixture 1:1 (50 mL) and neutralized with saturated K_2_CO_3_ solution. Then, the product was extracted with dichloromethane. Organic fractions were collected and evaporated to the dry green residue, which was chromatographed (silica gel, dichloromethane, and dichloromethane/methanol 50:1) to give **2** in the form of a deep green film (161 mg, 82% yield).

R*_f_* (dichloromethane/methanol 50:1) 0.52. UV-Vis (dichloromethane): λ_max_, nm (log *ε*) 730 (4.94), 655 (4.31), 436 (3.91), 321 (4.50), 256 (5.14). ^1^H NMR (800 MHz, Pyridine-*d*_5_) δ 7.93 (s, 8H), 7.92–7.88 (m, 16H), 6.93–6.90 (m, 16H), 5.17 (t, *J* 6.4 Hz, 16H), 4.30 (t, *J* 6.4 Hz, 16H), 4.18 (t, *J* 6.7 Hz, 16H), 2.56–2.51 (m, 16H), 2.38–2.34 (m, 16H), 1.56–1.51 (m, 16H), 1.32–1.25 (m, 16H), 0.82 (t, *J* = 7.4 Hz, 24H), 0.10 (s, 2H). ^13^C NMR (126 MHz, Pyridine-*d*_5_) δ 166.5, 163.7, 152.4, 132.1, 127.8, 123.3, 120.0, 114.9, 72.5, 68.8, 64.9, 31.4, 27.2, 27.1, 19.9, 14.3. MS (MALDI) *m*/*z* found: 2628.2361, [M + H]^+^ requires 2628.2619. HPLC purity: 96.48–100.00%.

#### 2.2.2. Zinc(II) 1,4,8,11,15,18,22,25-Octakis(4-[4-butoxycarbonylphenoxy]butyloxy)phthalocyanine (**3**)

Metalation reactions were performed by adapting a literature procedure [29]. Phthalocyanine derivative **2** (29 mg, 0.011 mmol) was dissolved in *N*,*N*-dimethylformamide (5 mL). Zinc(II) acetate (4 mg, 0.022 mmol) was added, and the reaction mixture was stirred for 20 h at 70 °C. After cooling, the solvent was evaporated under reduced pressure, and the green residue was chromatographed (silica gel, dichloromethane, then dichloromethane/methanol 50:1) to give **3** in the form of a deep green film (27 mg, 92% yield).

R*_f_* (dichloromethane/methanol 20:1) 0.20. UV-Vis (dichloromethane): λ_max_, nm (log *ε*) 810 (4.49), 741 (5.03), 666 (4.35), 329 (4.52), 256 (5.13). ^1^H NMR (800 MHz, Pyridine-*d*_5_) δ 8.03–7.99 (m, 16H), 7.92 (s, 8H), 7.01–6.98 (m, 16H), 5.26 (t, *J* 6.5 Hz, 16H), 4.28 (2 × t, *J* 6.6 Hz, 32H), 2.56–2.51 (m, 16H), 2.35–2.30 (m, 16H), 1.62–1.57 (m, *J* 6.7 Hz, 16H), 1.33 (m, 16H), 0.85 (t, *J* 7.4 Hz, 24H). ^13^C NMR (126 MHz, Pyridine-*d*_5_) δ 166.6, 163.7, 153.8, 152.5, 132.2, 130.0, 123.5, 119.8, 115.0, 72.9, 68.9, 65.0, 31.5, 27.2, 27.2, 19.9, 14.3. MS (MALDI) *m*/*z* found: 2692.1835, [M + H]^+^ requires 2692.1917. HPLC purity: 96.27–100.00%.

#### 2.2.3. Palladium(II) 1,4,8,11,15,18,22,25-Octakis(4-[4-butoxycarbonylphenoxy]butyloxy)phthalocyanine (**4**)

Phthalocyanine derivative **2** (79 mg, 0.030 mmol) was dissolved in *N*,*N*-dimethylformamide (5 mL). Bis(benzonitrile)palladium(II) chloride (23 mg, 0.060 mmol) was added, and the reaction mixture was stirred for 20 h at 70 °C. After cooling, the solvent was evaporated under reduced pressure, and the violet residue was chromatographed (silica gel, dichloromethane, dichloromethane/methanol 50:1, then hexanes, hexanes/ethyl acetate 7:5) to give **4** in the form of a deep green film (69 mg, 84% yield).

R*_f_* (dichloromethane/methanol 50:1) 0.19. UV-Vis (dichloromethane): λ_max_, nm (log *ε*) 728 (2.86), 652 (2.81), 440 (2.64), 322 (2.51), 256 (2.41). ^1^H NMR (800 MHz, Pyridine-*d*_5_) δ 7.93 (s, 8H), 7.82–7.79 (m, 16H), 6.88–6.85 (m, 16H), 5.23 (t, *J* 6.4 Hz, 16H), 4.31 (t, *J* 6.4 Hz, 16H), 4.11 (t, *J* 6.7 Hz, 16H), 2.57–2.51 (m, 16H), 2.38–2.33 (m, 16H), 1.51–1.46 (m, 16H), 1.28–1.22 (m, 16H), 0.80 (t, *J* 7.4 Hz, 24H). ^13^C NMR (101 MHz, Pyridine-*d*_5_) δ 166.2, 163.5, 152.07, 150.6, 142.9, 131.8, 128.4, 123.1, 119.8, 114.7, 72.6, 68.6, 64.7, 31.3, 27.1, 26.8, 19.8, 14.2. MS (MALDI) *m*/*z* found: 2734.1035, [M + H]^+^ requires 2734.1660. HPLC purity: 96.32–100.00%.

### 2.3. Emission Study

Fluorescence spectra were recorded with a Jasco 6200 spectrofluorometer. The measurements were performed in DMSO and DMF. Based on the collected data, fluorescence quantum yields were calculated following the previously described method [30,31,32,33]. Unsubstituted zinc(II) phthalocyanine (Sigma Aldrich, St. Louis, MO, USA) was used as the reference compound with known Φ_FL_.

### 2.4. Singlet Oxygen Generation Measurements

The analyses of the singlet oxygen generation quantum yields were carried out according to the previously described procedure in DMSO and DMF [30,31,34]. The wavelength was chosen accordingly to the absorption maximum in the Q-band determined separately for each compound. The experiments were performed under aerobic conditions at room temperature.

### 2.5. Photostability Determination

The selected compounds’ photodegradation analysis was performed according to the previously described protocols [30,35]. The assays were performed in DMSO and DMF. The light wavelength used for the experiments was narrowed down using a cutting filter (transmittance > 450 nm). The tests were performed under aerobic conditions at room temperature.

### 2.6. Lipid Vesicles Preparation

All phthalocyanines, due to their limited solubility in water, were incorporated into liposomes consisting of commercially available phospholipids POPC (1-palmitoyl-2-oleoyl-sn-glycero-3-phosphocholine) and DOTAP (N-[1-(2,3-dioleoyloxy)propyl]-*N*,*N*,*N*-trimethylammonium chloride, 25 mg/mL) provided by Avanti Polar Lipids Inc. The formulations were prepared accordingly to a modified procedure described by Dragicevic-Curic et al. [36]. In brief, lipids dissolved in chloroform were placed in a tube in an 8:2 molar ratio with a proper amount of PS solutions in chloroform and mixed. Further, the solvent was completely evaporated under reduced pressure at room temperature. The obtained lipid film was treated with sterile phosphate-buffered saline (PBS) and vortexed for 10 min (1500 RPM) to form liposomes. The final formulation was stored at 2–4 °C in the dark. The size of the liposomes was determined using a NanoSight LM10 (Malvern Panalytical, Malvern, UK).

### 2.7. Antimicrobial Activity

#### 2.7.1. Microbial Cultures

The following microbes were used in the experiment: methicillin-resistant *Staphylococcus aureus* (*MRSA*) (clinical strain), beta-lactamase-producing *Escherichia coli* (clinical strain), *Candida auris* (DSM 21092), *Candida albicans* fluconazole-resistant (ATCC 10231), *Trichophyton rubrum*, and *Trichophyton mentagrophytes* (ATCC 9533). *MRSA* and *E. coli* (ESBL+) were cultured in a brain heart infusion broth (bioMerieux, Marcy-l’Étoile, France) for approximately 20 h at a constant temperature of 35 ± 1 °C under aerobic conditions. *C. albicans* and *C. auris* were cultivated for approximately 24 h at 35 ± 1 °C in the Sabouraud dextrose broth (Oxoid, Hampshire, UK) and Emmons’ modification of Sabouraud’s broth (BD), respectively. After the incubation period, the bacteria and fungi were centrifuged and harvested (3000 rpm for 15 min) and then re-suspended in 10 mM phosphate-buffered saline (PBS, pH = 7.0). *T. rubrum* and *T. mentagrophytes* in Sabouraud dextrose agar (Oxoid, Hampshire, UK) at 35 ± 1 °C until adequate sporulation appeared (ca. three weeks). After incubation, cultures were covered with sterile 0.9% NaCl solution supplemented with 0.1% Tween 80, carefully rubbed with a sterile cotton swab, and transferred to a sterile flask. Suspensions were homogenized and filtered. In the last step, each strain was adjusted at a concentration of ca. 10^7^ CFU/mL.

#### 2.7.2. Dark Activity

In the first step, MIC tests were performed for each compound and strain according to the procedure described by Wiegand et al. (the dilution method) [37]. Twice the highest value used in the dark toxicity study was utilized as the initial concentration. In the second step, the appropriately prepared microbial suspension was placed on a 96-well microtiter plate. A liposomal phthalocyanine formulation was added to each well. A control sample was prepared analogously, but the PS was replaced with PBS. The plate was then placed in a laboratory shaker (speed 60 rpm) for 30 min in the dark. After the incubation period, a sample of microorganisms was collected from each well and then plated after dilution. *MRSA* and *E. coli* (ESBL+) were grown for approximately 24 h at 35 ± 1 °C on tryptic soy agar (TSA) plates. *C. albicans*, *T. menagrophytes* and *T. rubrum* were spread on Sabouraud agar plates, while *C. auris* was spread on Emmons’ modification of Sabouraud agar plates. *C. albicans* and *C. auris* were incubated on the plates for about 72 h, and dermatophytes for about 5–7 days. The number of viable microorganisms was calculated by counting the number of colony-forming units (CFUs). Based on the results, the log reduction of the microorganisms was determined. Experiments were performed in triplicates.

#### 2.7.3. Light-Dependent Activity

Analogously to the dark activity, the light activity of the Pc formulation was evaluated. The previously prepared bacterial suspension was placed in a 96-well plate, and then the liposomal phthalocyanine formulation was added. For the control, PBS was added instead of the PS, and then incubated in a laboratory shaker for 30 min (speed 60 rpm) in the dark. After this time, the plate was illuminated with light in the range of 730–740 nm emitted by LEDs panel (epiLED, Wroclaw, Poland) with a total light dose of 50 J/cm^2^ or 100 J/cm^2^. The number of living organisms was evaluated according to the scheme described above. The experiment was repeated in triplicate.

#### 2.7.4. Determination of Microorganism’s Susceptibility to PACT and Antibiotics Following Habituation with Sub-Lethal PACT

According to the protocol established by Cassidy et al. [38] for each of the phthalocyanine derivatives, the dose resulted in a reduction in the range of 0.5–1.0 log (sublethal dosimetry of PS) was determined. The procedure was repeated separately for each strain of bacteria and fungi. Weakened cultures were treated with antibiotics to determine the selected antibiotics’ minimum inhibitory concentration (MIC). The antibiotic susceptibility test was performed using the dilution method according to the procedure described by Wiegand et al. [37]. *MRSA* was treated with meropenem and ceftriaxone, *E. coli* (ESBL+) with ceftriaxone and gentamicin, and fungi and dermatophytes were treated with ciclopirox and terbinafine. In parallel, a controlled trial was carried out on cultures not subjected to PACT. Each test was carried out in triplicate.

### 2.8. Statistical Analysis

The data represent the mean from the experiment performed in triplicate. The unpaired Student’s *t*-test and U Mann–Whitney test were used to establish the significance of differences between groups. A probability value (*p*) of less than 0.05 was considered significantly different. Statistical analysis was performed with the STATISTICA software, v.13.0.

## 3. Results and Discussion

### 3.1. Synthesis

**Scheme 1:**
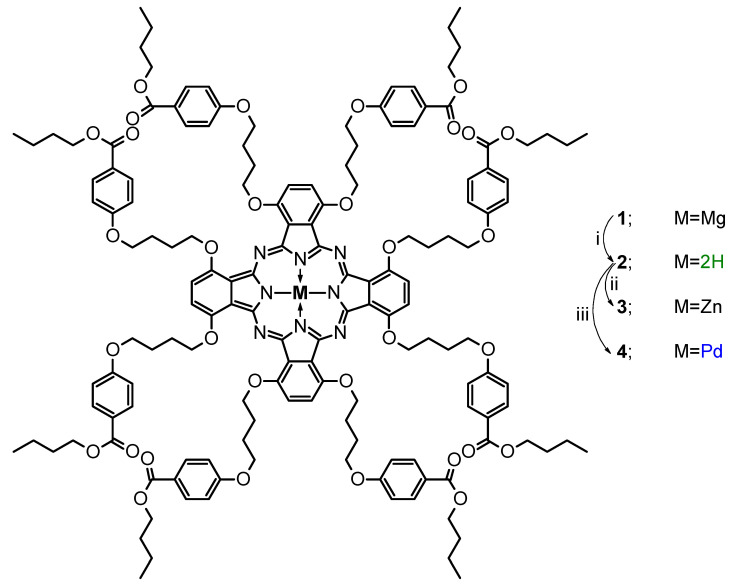
Synthesis of phthalocyanine derivatives **2**–**4**. Reaction conditions: (i) trifluoroacetic acid, room temperature, 30 min; (ii) zinc(II) acetate, dimethylformamide, 70 °C, 20 h; (iii) bis(benzonitrile)palladium(II) chloride, dimethylformamide, 70 °C, 20 h.

The starting material for the synthesis of novel Pc derivatives was lately studied magnesium(II) 1,4,8,11,15,18,22,25-octakis(4-[4-butoxycarbonylphenoxy]butyloxy)phthalocyanine (**1**) [23]. Pc derivative **1** was subjected to the demetalation reaction with trifluoroacetic acid over 30 min at room temperature (Scheme 1). After the workup and the purification of the reaction mixture, a free base macrocycle **2** was obtained with 82% yield.

Next, Pc derivative **2** was subjected to metalation reactions by heating at 70 °C in dimethylformamide with zinc(II) acetate or bis(benzonitrile)palladium(II) chloride towards macrocycles **3** or **4**, respectively (Scheme 1). After chromatographic purification, Pc derivatives were obtained with high yields, **3** with 92% and **4** with 84%.

All the newly obtained compounds were subjected to physicochemical characterization, including UV-Vis and NMR spectroscopy (^1^H NMR, ^13^C NMR and 2D techniques—^1^H-^1^H COSY, ^1^H-^13^C HSQC, and ^1^H-^13^C HMBC), as well as mass spectrometry. In the ^1^H NMR spectrum of **2**, a characteristic signal at 0.10 ppm resulting from the presence of NH group hydrogen atoms in the macrocycle core is observed. The aliphatic proton signals were assigned based on the 2D experiments with butyl substituents signals appearing at 0.82, 1.29, 1.54, and 4.18 ppm corresponding to CH_3_, CH_2_, CH_2,_ and CH_2_O protons, respectively. The aliphatic proton signals of the butylene linker were noted at 4.30 (CH_2_O), 2.36 (CH_2_), 2.54 (CH_2_), and 5.17 (CH_2_O) ppm, with the latter CH_2_O group being attached to the macrocyclic ring. In the aromatic region of the spectrum, three signals are present, of which singlet at 7.93 ppm corresponds to the Pc ring protons. In contrast, the other two signals at 7.90 and 6.91 ppm were assigned to the aromatic hydrogens present in the nipagin-functionalized peripheral groups. In the ^1^H NMR spectra recorded for **3** and **4**, there are no significant changes present compared to the **2** spectrum—the most obvious is the lack of the 0.10 ppm signal. Proton signals present in the ^1^H NMR spectra of **3** and **4** are slightly up- or downfield shifted as compared to the signals present in the spectrum of the metal-free derivative. Butyl substituent signals present in the spectrum of Pc derivative **3** are observed at 0.85, 1.33, 1.59, and 4.28 ppm, whereas the butylene linker hydrogen atoms signals appear at 4.28, 2.33, 2.54, and 5.26 ppm. In the ^1^H NMR spectrum of **4**, the corresponding signals of butyl substituent and butylene linker can be found at 0.80, 1.26, 1.48, 4.11 ppm, and 4.31, 2.35, 2.54, 5.23 ppm. The signals from the hydrogen atoms in the *para*-substituted benzene ring (nipagin) are present at 8.01 and 6.99 ppm for **3** and 7.81 and 6.89 ppm for **4**. The Pc ring protons in peripheral positions appear as singlets at 7.92 ppm for **3** and 7.93 ppm for **4**.

### 3.2. Spectral Properties

**Figure 1:**
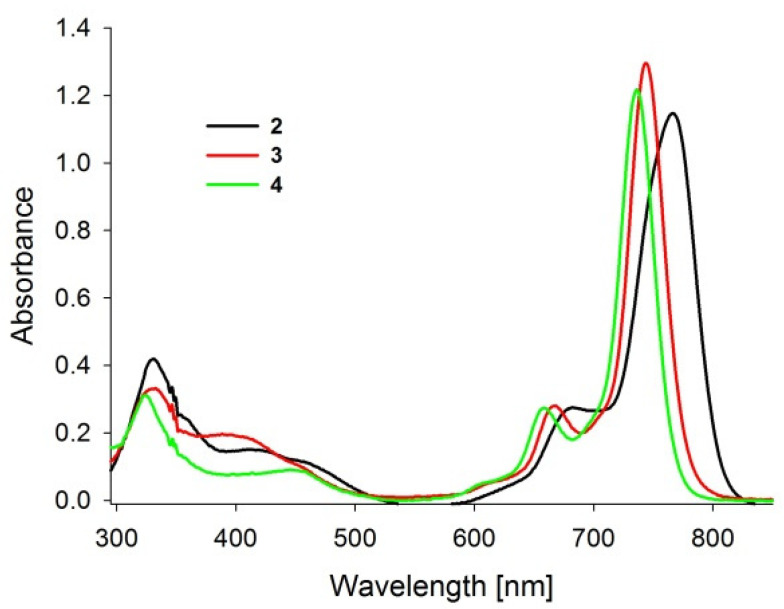
Absorption spectra of phthalocyanines **2**–**4** in dimethyl sulfoxide (concentration range 6.9–8.7 × 10^−6^).

The recorded absorption spectra of studied nipagin-functionalized Pcs revealed typical absorption bands, when compared to that of previously studied Pcs (Figure 1) [23,39,40].

The Soret band is a result of π–π* electron transitions from molecular orbitals to the low unoccupied molecular orbital. The Soret band maxima of herein studied PSs are placed at ca. 330 nm. The Q band wavelength ranges of Pcs **2**–**4** are located between 650–810 nm. The appearance of the Q band is a result of π–π* electron transitions from the highest occupied molecular orbital to the degenerated unoccupied molecular orbital. The absorption maximum for a demetalated Pc **2** appears in DMF and DMSO at 765 nm. The value is similar to that reported before for structurally similar demetalated derivative [41]. Zinc(II) phthalocyanine derivative **3** revealed the Q band maxima at 739 nm and 744 nm in DMF and DMSO, respectively. The Q band maxima of palladium(II) Pc derivative **3** are located at 733 nm in DMF and 736 nm in DMSO. It could be noted the hypsochromic shift of the Q bands of metalated Pcs in comparison to the demetalated ones. Based on previous research, it can be explained by the destabilization of a low unoccupied molecular orbital and reflects the orbital interactions between the ligand and zinc or palladium cations in cores [42,43]. Molar absorption coefficients of studied compounds are typical for Pcs with logε up to 5.25, and comparable to the earlier reported palladium(II) and zinc(II) Pc derivatives [44,45].

**Figure 2:**
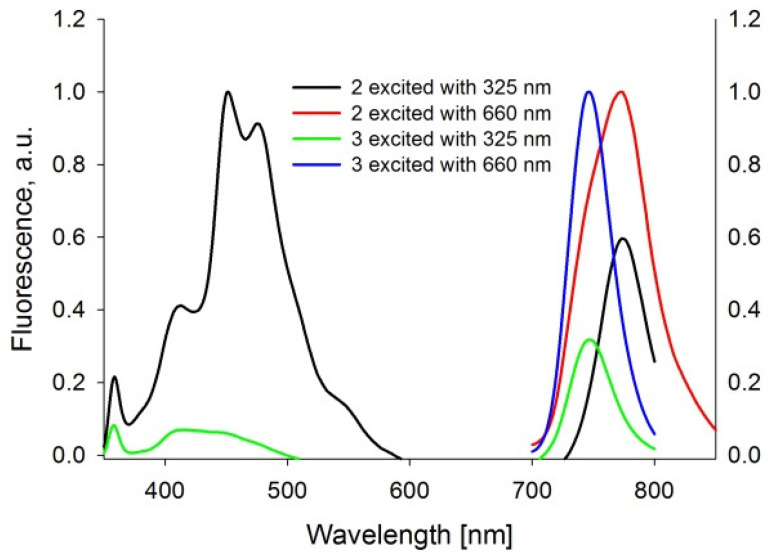
Fluorescence spectra of **2** and **3** in DMF solutions.

The fluorescence properties of PSs were recorded and analyzed (Figure 2). In the emission spectra of zinc(II) Pc (**3**), the demetalated Pc (**2**) were recorded in DMF and DMSO after the excitation at 325 nm and 660 nm. Two emission bands were noted after the excitation of **2** and **3** dissolved in DMF in the absorption Soret band regions at 325 nm. The first emission is in the blue region and reflects the emission from the S_2_ excited state, whereas the second one in the red region corresponds to the emission from the S_1_ excited state (Figure 2). This kind of emission is called a dual emission following previous reports [46,47,48,49]. Both fluorescent compounds **2** and **3** showed a low quantum yield of fluorescence at the same level Φ_FL_ equal to 0.03 in DMF, whereas, in DMSO, no emission band was noted for **2** (Table 1). As could be expected, a palladium(II) Pc derivative (**4**) did not reveal any fluorescence due to the efficient formation of the excited triplet states. This fact was described before for various palladium complexes [50] and is related to the palladium(II) ion being a closed shell ion [51]. The influence of the coordinated zinc(II) cation on the electron configuration of the macrocycle is reflected in the emission spectra of **3**, where the blue emission band reveals lower intensity in comparison to the same band for **2**. The scale of the blue band decay can be related to the spin-orbit coupling effect, also responsible for the formation of excited states. Spin-orbit coupling is higher for larger ions [51]. Therefore, we observed a limited emission in the blue region for **3** and lack of emission for **4**.

### 3.3. Photostability Studies

The stability of PS under irradiation constitutes a critical parameter to consider when dosimetry is planned in experiments in vitro, in vivo, and clinical trials. Therefore, the photostability properties of Pcs **2**–**4** were studied. High pressure xenon lamp with defined emission spectrum has been used in the experiment (see Supplementary Material). Measurements have been performed at ambient temperature and air conditions.

**Figure 3:**
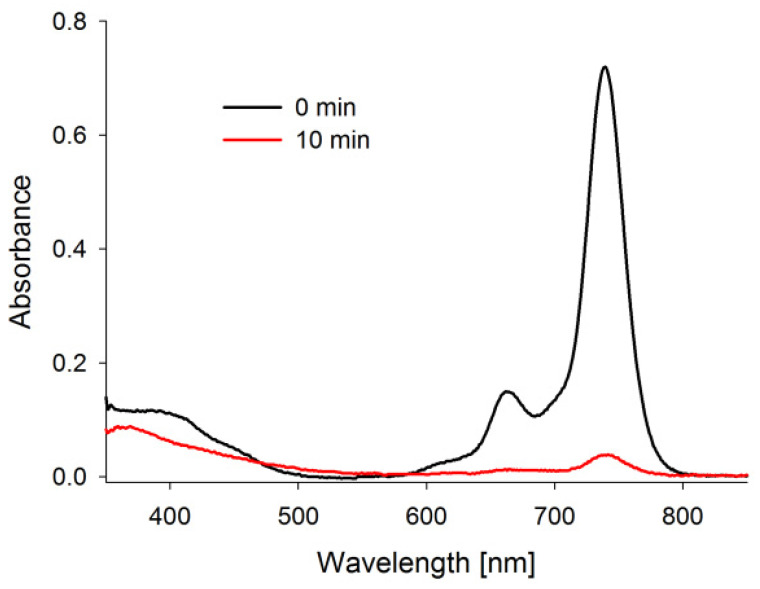
Changes in the spectra during irradiation of **3** in DMF after irradiation with visible light (>450 nm).

Macrocycles **2**–**4** underwent photodecomposition processes during irradiation, which was manifested in a loss of color of the samples. In the absorption bands, the short wavelengths Soret bands changed intensity and profile after irradiation (Figure 3), which could be linked to a low-weight photoproducts formation. The changes in the absorption of the Q band ranges after irradiation revealed a negligible intensity, which could be explained by an incomplete photodegradation process occurring in the studied conditions. Considering the above, it can be concluded that decomposition of the studied Pcs followed by visible light irradiation proceeds via a photobleaching process. Interestingly, **3** and **4** as efficient singlet oxygen generators showed a high photostability. In the case of **1** [23] and **3**, the chosen solvent—DMF or DMSO, affected the increase in photostability. In the DMSO solutions, solvent coordinates to the central metal ion and in this way stabilizes the molecule [55]. Palladium (II) complex **4** revealed equivocal properties and showed the highest photostability and singlet oxygen formation ability. It is interesting because for some palladium(II) Pcs described in the literature, only low photostability was reported with photodecomposition quantum yield (Φ_P_) at the level of 10^−4^ [56]. Following literature data, PS with Φ_P_ equal to ca. 10^−6^ is treated as photostable, whereas PS with Φ_P_ equal to ca. 10^−3^ is classified as photolabile [57]. The calculated Φ_P_ values (Table 1) allow classifying the studied PSs as photostable or with moderate stability after irradiation with light.

### 3.4. Singlet Oxygen Formation

**Figure 4:**
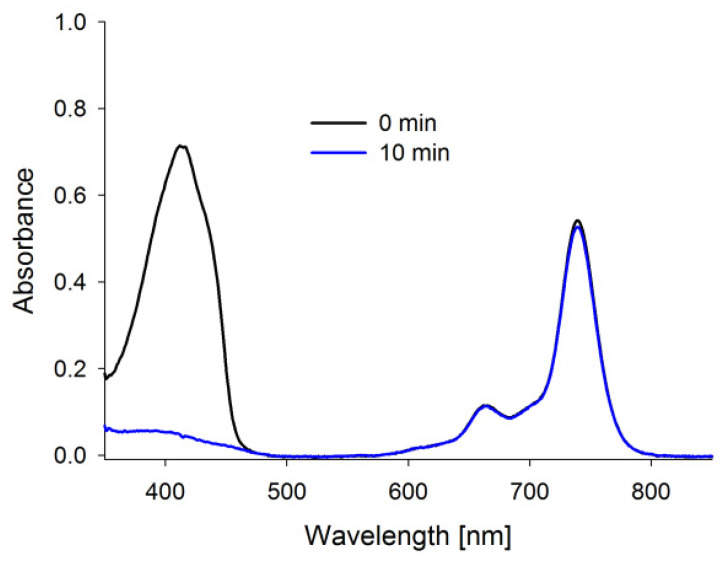
Absorption spectra of phthalocyanine derivative **3** and DPBF mixture in dimethylformamide at the beginning (0 min) and after 10 min of irradiation (power density: 0.5 mW/cm^2^ at 740 nm).

Pc derivatives **2**–**4** were also assessed in terms of singlet oxygen (^1^O_2_) production potential using a comparative method with 1,3-diphenylisobenzofuran (DPBF). In the method, singlet oxygen produced during the irradiation of PS is quenched by DPBF, which can be monitored in the UV-Vis by the disappearance of the characteristic absorption band with a maximum at ca. 417 nm (Figure 4). It is worth noting that singlet oxygen is a dominant agent combating bacteria in a photodynamic reaction [2]. All studied compounds **2**–**4** revealed potential for singlet oxygen generation under irradiation with light at a wavelength corresponding to their Q band maxima. Pc **2**, due to the lack of metal ion in the coordination center was the weakest singlet oxygen generator with a quantum yield up to 0.10. The introduction of the zinc(II) and palladium(II) ions to the Pc core significantly impacted the formation of singlet oxygen quantum yields (Φ_∆_). It can be justified by the nature of metal cations with closed shells, which are known to improve this parameter [51]. Zinc(II) Pc complex (**3**) revealed Φ_∆_ equal to 0.55 in DMF and 0.72 in DMSO, which corresponds to the literature’s values for the unsubstituted zinc(II) Pc, which was used as a reference. This also allows concluding that non-peripheral substituents did not significantly affect the singlet oxygen production ability. In the case of a palladium(II) complex **4,** further increase of Φ_∆_ (up to 0.77) was observed. It is linked to a spin-orbit coupling effect, which is more efficient for larger cations with complex electron structures, especially with d and f orbitals [51]. This regularity can be confirmed by comparing a magnesium(II) Pc complex with the herein studied zinc and palladium ones. Our previous study found that the closed-shell metal cation present in the core of Pc **1** allowed to form ^1^O_2_ with quantum yields of 0.29 in DMF and 0.13 in DMSO [23]. High Φ_∆_ values for palladium(II) macrocyclic complexes were also reported in other studies [56,58,59,60].

### 3.5. Liposome Vehicles

As most PSs from the porphyrinoid group, herein studied Pcs **2**–**4** revealed a highly hydrophobic character. Therefore, they were incorporated into liposomes to allow in vitro studies. Liposomes also reveal a potential for further in vivo trials.

Liposomes are vehicles with a specific structure characterized by a hydrophobic interior covered with hydrophilic headgroups from both sites of a spherically shaped membrane. They consist mainly of phospholipids and could possess additional ingredients modifying their structure. Moreover, liposomal formulations can reduce PS aggregation and maintain their photosensitizing efficacy [61]. Hydrophobic drugs locate inside the membrane during liposome formation [7]. Liposome formulations of Pcs **2**–**4** have a diameter mean of 229.6 nm, 161.5 nm, and 254.4 nm, respectively. The size of the formed vehicles is regulated among others by the nature of an enclosed compound [62]. The potential application of the developed formulations in intravenous administration is excluded due to the size limit, but independently they appear suitable for skin disorders treatment [63,64,65,66].

### 3.6. Antimicrobial Photodynamic Activity

Photoactivity of the obtained Pcs **2**–**4** encapsulated in a liposomal formulation were studied against a broad spectrum of microorganisms, including Gram-positive bacteria MRSA, Gram-negative *E coli* (ESBL+), fungi *C. albicans* (fluconazole-resistant), *C. auris* and dermatophytes *T. mentagrophytes* and *T. rubrum*. In addition, specific issues were also studied, including the influence of the exposure time, the concentration of PSs, as well as the bactericidal effect. No growth inhibition was observed with any test strain or compound without irradiation (MIC outside the range of the study, see Supplementary Material). Even using a concentration twice higher than in the irradiated test, no bactericidal effect was observed. In view of these results, no IC_50_ determination was attempted. In the study, the demetalated Pc **2** turned out to be unambiguously the least active, whereas phthalocyanines **3** and **4** revealed attractive photocytotoxic potential towards microorganisms. It is worth noting that palladium(II) Pc derivative **4** revealed higher bactericidal properties than zinc(II) Pc **3**. This phenomenon has already been observed in other studies, especially photocytotoxicity studies on cancer cell lines, and was associated with the generation singlet oxygen quantum yield. The increase of Φ_∆_ was correlated directly with the efficacy of PDT against HeLa cells [60].

Compound causing bacterial growth reduction of about 3 log could be classified as bactericidal [67]. Some authors have established even 4 logs as a bactericidal border [68]. Among all herein studied PSs, palladium(II) Pc derivative **4** was assessed as bactericidal against *MRSA*. More than 4 log reductions against *MRSA* at both lower (10 µM) and higher (100 µM) concentrations have been noted after activation with 50 J/cm^2^ light. When the light dose was raised to 100 J/cm^2^, some slight reduction of the photocytotoxicity of **4** against *MRSA* was observed. However, the mentioned changes reveal any clinical significance, they should be considered when planning the dosimetry protocol. It is well-known that a higher light dose could cause pain during irradiation [69]. Pc derivative **3** approached the bactericidal border of 3 log (2.72 log) at the concentration of 100 µM and the light dose 100 J/cm^2^ for the reduction of the *MRSA* (Table 2).

In the case of Gram-negative *E. coli* (ESBL+) bacteria, the bactericidal effect of **4** at 5.22 log was observed only at the higher concentration (100 µM) and after the irradiation with a lower light dose (50 J/cm^2^). An increase in the light dosimetry caused an improvement in results to the level above 5.78 log. At the high concentration and high light dose, PSs **2** and **3** presented equally high activity up to >4.90 log. For Pc derivatives **2** and **3** more potent light dose was applied to achieve a high activity. It was related to the lower Φ_∆_ of these two PSs in comparison to Pc **4**.

**Figure 5:**
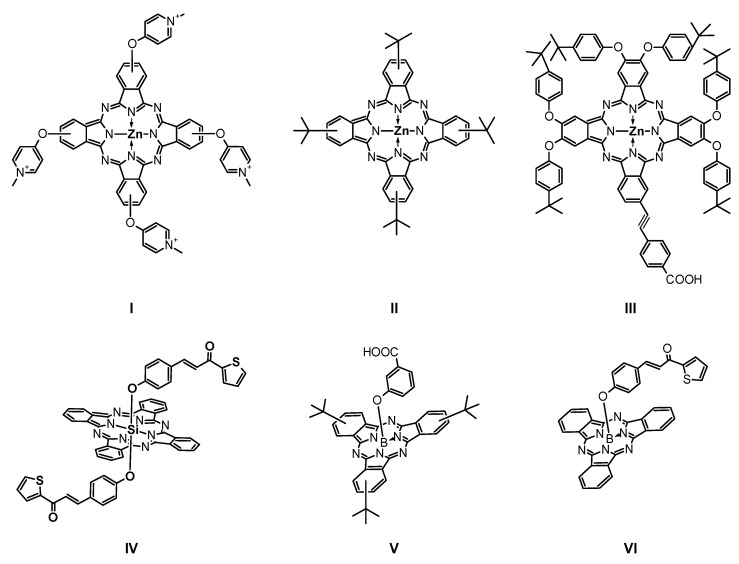
Chemical structures of phthalocyanine (**I**–**IV**) and subphthalocyanine (**V**–**VI**) derivatives.

Surprisingly, studied Pcs revealed a high photocytotoxic potential against fluconazole-resistant *C. albicans* (Table 2). Among all studied Pcs, phthalocyanine derivative **2** under the red-light irradiation at the dose of 50 J/cm^2^ and a concentration of 100 µM reduced *C. albicans* by 5.73 log. Equally promising results were given by the same PS at a lower concentration but subjected to a higher light dose. Zinc(II) Pc derivative **3** turned out to be highly effective against *C. albicans* in a concentration of 10 µM and a light dose of 50 J/cm^2^. The increase in the PS concentration did not change the activity significantly. Similarly, the higher light dose did not significantly improve bacterial growth reduction (Table 2). The obtained results of the combat fluconazole-resistant *C. albicans* are promising in comparison to the studies published so far. In two experiments conducted by Palma et al. zinc(II) Pc derivative (**I**, Figure 5) at the concentration of 10 µM and 5 µM revealed effectivity against *C. albicans* by presenting ca. 5 log reduction [70,71]. In the photocytotoxic study on *Candida* performed by Ozturk et al., zinc(II) Pc and subphthalocyanine derivatives (**II**–**VI**, Figure 5) at the concentration of 128 µg/mL reduced growth by ca. 6 log [72].

In all the above-presented studies, the strains were not defined as resistant to fluconazole. It should be noted that in the case of most of the PSs evaluated before, their photocytotoxicity against bacteria has not correlated with the efficacy against *C. albicans* [73,74,75,76]. Even though **4** revealed high singlet oxygen generation properties, its photoinactivation potential against *C. albicans* was lower than that presented by **3** and comparable to **2**. The highest photocytotoxicity at 4.88 log was noted against fluconazole-resistant *C. albicans* by **4** at the concentration of 10 µM and 50 J/cm^2^ of light. By increasing the light dose, an improvement of the reduction potential above 5.78 log was noted.

Promising results in the photodynamic inactivation of *C. albicans* prompted us towards further experiments with studied Pcs against *Candida auris*. *C. auris* is a pathogen that has recently been identified as a hazard to humans. It was isolated for the first time in 2009 from a patient’s ear in Japan [77]. This fungus is classified as multidrug-resistant due to its insensitivity to most used antibiotics. An additional threatening factor is the ability of *C. auris* to survive both on usable surfaces and the human body [78]. PDT is exceptionally effective in the case of superficial infections. To date, limited data are available about the possibility of the PACT use against *C. auris*. In an experiment conducted by Tan et al., methylene blue (MB) at the concentration of 8 µg/mL revealed a reduction of approximately 3 log [79]. It should be emphasized that MB belongs to the oldest generation of PS and has many limitations. The limitations mentioned above about MB include, first of all, its ability of removal from the cell via an efflux-pump mechanism by some microorganisms, and its tendency to aggregation or self-degradation [1,2,3]. Bapat and Nobile have analyzed the effectiveness of the new methylene blue, rose bengal, and toluidine blue against *C. auris* biofilms. Depending on the source of the light used, up to 85% (<1 log reduction in fungi growth) inhibition was achieved [1]. In our studies, the best potential in *C. auris* inactivation was noticed for Pc derivative **3** (>5.05 log) at a concentration of 100 µM and after excitation with 100 J/cm^2^ light dose. Interestingly, PS **2** revealed a similar photodynamic activity against *C. auris* to **4** at the same dosimetry parameters (100 µM, 100 J/cm^2^). It should be pointed out that Pc derivative **2** is the weakest ^1^O_2_ generator with Φ_∆_ equal to 0.10, whereas **4** is the best with Φ_∆_ equal to 0.77. At the lower concentrations and light doses studied PS group was inactive against this pathogen. The results present the opportunity to develop a highly efficient therapy against *C. auris*, predominantly when these fungi infect the earlobe, which can be efficiently irradiated.

An interesting paradox can be noted in the case of PACT activity against *C. auris*. In contrast to *C. albicans*, most PSs are active even at lower concentrations. *C. auris* presents limited inborn sensitivity to a number of antifungal drugs, including azoles, echinocandins, and polyenes [80]. The production of such a wide range of resistance mechanisms forces the cell to increase metabolism. For *C. auris* strain, a higher activity of ABC and MFS efflux pump is observed, as well as structural changes in the cell wall are present [80]. The metabolic effort directed into these defense pathways could increase the sensitivity for the pathogen towards PACT. This phenomenon has not yet been fully researched and understood, but the so-called increased sensitivity (collateral sensitivity) has been so far observed, among others, during the studies performed with antimicrobial peptides [81,82].

Further studies were focused on dermatophytes *T. rubrum* and *T. mentagrophytes*. Only PS **3** was found to effectively combat *T. rubrum* at 3.7 log at the higher concentration and after irradiation with a light of 100 J/cm^2^. Other studied PSs showed much lower activity (Table 2). Simultaneously, no molecule from the evaluated PSs was active against *T. mentagrophytes*.

The synergy between PSs **2**–**4** and commonly used antibiotics and antifungal drugs was also studied. In the case of fungi and dermatophytes, terbinafine and ciclopirox were used; against *E. coli* (ESBL+), gentamicin and ceftriaxone; and against *MRSA*, meropenem and ceftriaxone. There was no clinically significant change in the MIC value for each PS-antibiotic combination. In the light of the results conducted so far, the critical aspect of this failure may be the chemical structure of studied PSs, which were functionalized in non-peripheral positions with nipagin substituents. Despite many years since the introduction of nipagins to the market, there is no clear explanation of their mechanism of action [83]. One possible pathway is the disruption of cellular membrane transfer processes [84]. It seems that nipagin-functionalized Pcs do not bind to or pass the bacterial membrane as nipagin molecules act as individual molecules. Thus, no synergism between the sub-lethal PACT and antibiotic therapy was observed.

## 4. Conclusions

Three Pcs functionalized with nipagin-substituents at non-peripheral positions were obtained starting from the previously synthesized magnesium(II) 1,4,8,11,15,18,22,25-octakis(4-[4-butoxycarbonylphenoxy]butyloxy)phthalocyanine. Magnesium(II) Pc derivative was subjected to the demetalation reaction in trifluoroacetic acid, and subsequently used in the metalation reaction with dimethylformamide with zinc(II) acetate and bis(benzonitrile)palladium(II) chloride towards zinc(II) and palladium(II) derivatives. Pc derivatives were characterized using 1D and 2D NMR spectroscopy and mass spectrometry, as well as subjected to absorption, emission, and photostability studies. Zinc(II) and palladium(II) Pc complexes in the photochemical study in DMF and DMSO revealed high quantum yields of singlet oxygen generation from 0.55 to 0.77. Liposomal formulations of Pc derivatives were prepared, and their activity was evaluated against a broad spectrum of antibiotic-resistant microorganisms, such as Methicillin-resistant *S. aureus* (*MRSA*), *E. coli* (ESBL+), *C. albicans* resistant to fluconazole, *C. auris*, and against dermatophytes. The dependencies between the exposure time, the concentration of PSs, and the bactericidal effects were also studied. A clear relationship was observed between the presence of zinc(II) or palladium(II) metal ions in the core of the macrocycles and their photocytotoxicities against tested microorganisms. The demetalated Pc turned out to be unambiguously the least active. Such a substantial difference was not detected between zinc(II) and palladium(II) Pc derivatives. However, palladium(II) Pc derivative was noticeably more bactericidal than zinc(II) Pc. More than 4 log growth reduction of *MRSA*, *E. coli* (ESBL+), *C. albicans* fluconazole-resistant in the presence of studied PSs was noted. It should be underlined that for the zinc(II) Pc derivative, over 5.05 log reduction of *Candida auris* growth was noted. Interestingly, the Pc palladium(II) complex showed the highest bactericidal activity against all antibiotic-resistant microorganisms, including a 3.54 log reduction of *C. auris*. Unfortunately, there was no synergy between the nipagin-functionalized Pcs and commonly used antibiotics.

## Figures and Tables

**Table 1:** Quantum yields of fluorescence, photodecomposition, and singlet oxygen formation of phthalocyanines **2**–**4**.

Compound	Solvent	Φ_FL_	10^6^ Φ_P_	Φ_Δ_
**2**	DMF	0.03	51.86	0.03
	DMSO	-	46.33	0.10
**3**	DMF	0.03	70.04	0.55
	DMSO	0.01	2.29	0.72
**4**	DMF	-	9.03	0.73
	DMSO	-	1.35	0.77
**ZnPc**	DMF	0.20 [52]	10.20 [53]	0.56 [54]
	DMSO	0.17 [52]	3.50 [53]	0.67 [54]

**Table 2:** Biological activity of phthalocyanine derivatives **2**–**4** against various microorganisms.

	*MRSA*		*E. coli (*ESBL+*)*		*C. albicans* (Fluconasole Resistant)		*C. auris*		*T. mentagrophytes*	*T. rubrum*
Concentration [M]	10^−4^	10^−5^	10^−4^	10^−5^	10^−4^	10^−5^	10^−4^	10^−5^	10^−5^	10^−5^
Light Dose [J/cm^2^]	Log Reduction in Bacterial Growth									
**2**										
50	0.35	0.33	2.53	2.13	5.73	0.63	n.a.	n.a.	n.a.	2.12
100	2.26	0.52	>4.9	1.13	5.48	5.78	3.59	0.89	n.a.	1.60
**3**										
50	2.30	0.99	2.53	0.36	4.50	5.08	n.a.	0.46	n.a.	2.11
100	2.72	1.56	4.28	1.95	5.78	>5.78	>5.05	0.76	n.a.	3.70
**4**										
50	4.39	3.99	>4.92	0.75	4.88	5.22	n.a.	0.17	n.a.	2.71
100	3.51	3.16	3.88	2.17	5.48	>5.78	3.54	1.45	n.a.	2.71

n.a.—no activity.

## Data Availability

Not applicable.

## Supplementary Materials

The following supporting information can be downloaded at: https://www.mdpi.com/article/10.3390/pharmaceutics14081686/s1, Table S1: ^1^H and ^13^C NMR data were obtained for 2 including key correlations determined from ^1^H-^1^H COSY, ^1^H-^13^C HSQC and ^1^H-^13^C HMBC spectra. Figure S1: ^1^H and (^13^C) chemical shift values [ppm] and key correlations observed in NMR spectra of 2. Bold lines: ^1^H-^1^H COSY; Arrows: ^1^H-^13^C HMBC. Figure S2: ^1^H NMR spectrum of 2 (800 MHz, pyridine-d_5_, 298 K). The symbols * and ~ indicate pyridine-d_5_ and water residual peaks, respectively. Figure S3: ^13^C NMR spectrum recorded for 2 (126 MHz, DMSO-d_6_, 298 K). The symbol * indicates DMSO-d_6_ residual peak. Table S2: ^1^H and ^13^C NMR data were obtained for 3 including key correlations determined from ^1^H-^1^H COSY, ^1^H-^13^C HSQC and ^1^H-^13^C HMBC spectra. Figure S4: ^1^H and (^13^C) chemical shift values [ppm] and key correlations observed in NMR spectra of 3. Bold lines: ^1^H-^1^H COSY; Arrows: ^1^H-^13^C HMBC. Figure S5: ^1^H NMR spectrum of 3 (800 MHz, pyridine-d_5_, 298 K). The symbols * and ~ indicate pyridine-d_5_ and water residual peaks, respectively. Figure S6: ^13^C NMR spectrum recorded for 3 (126 MHz, pyridine-d_5_, 298 K). The symbol * indicates pyridine-d_5_ residual peak. Table S3: ^1^H and ^13^C NMR data obtained for 4 including key correlations determined from ^1^H-^1^H COSY, ^1^H-^13^C HSQC and ^1^H-^13^C HMBC spectra. Figure S7: ^1^H and (^13^C) chemical shift values [ppm] and key correlations observed in NMR spectra of 4. Bold lines: ^1^H-^1^H COSY; Arrows: ^1^H-^13^C HMBC. Figure S8: ^1^H NMR spectrum of 4 (800 MHz, pyridine-d_5_, 298 K). The symbols * and ~ indicate pyridine-d_5_ and water residual peaks, respectively. Figure S9: ^13^C NMR spectrum recorded for 4 (126 MHz, pyridine-d_5_, 298 K). The symbol * indicates pyridine-d_5_ residual peak; HPLC DATA sheets for 2,3,4. Figure S10. Chromatogram of 2 in phases configuration 1. Table S4. Separation conditions and parameters of obtained signals for 2 in phases configuration 1. Figure S11. Chromatogram of 2 in phases configuration 2. Table S5. Separation conditions and parameters of obtained signals for 2 in phases configuration 2. Figure S12. Chromatogram of 2 in phases configuration 3. Table S6. Separation conditions and parameters of obtained signals for 2 in phases configuration 3. Figure S13. Chromatogram of 3 in phases configuration 1. Table S7. Separation conditions and parameters of obtained signals for 3 in phases configuration 1. Figure S14. Chromatogram of 3 in phases configuration 2. Table S8. Separation conditions and parameters of obtained signals for 3 in phases configuration 2. Figure S15. Chromatogram of 3 in phases configuration 3. Table S9. Separation conditions and parameters of obtained signals for 3 in phases configuration 3. Figure S16. Chromatogram of 4 in phases configuration 1. Table S10. Separation conditions and parameters of obtained signals for 4 in phases configuration 1. Figure S17. Chromatogram of 4 in phases configuration 2. Table S11. Separation conditions and parameters of obtained signals for 4 in phases configuration 2. Figure S18. Chromatogram of 4 in phases configuration 3. Table S12. Separation conditions and parameters of obtained signals for 4 in phases configuration 3. Figure S19. High preasure xenon lamp emission. Figure S20. Dark toxicity of studied compounds against bacteria and fungi.
