# Supplementary material for: Zinc(II), Palladium(II), and Metal-Free Phthalocyanines Bearing Nipagin-Functionalized Substituents against Candida auris and Selected Multidrug-Resistant Microbes
Source: Pharmaceutics. 2022 Aug 12;14(8):1686. doi: 10.3390/pharmaceutics14081686 (PMC9416722; doi:10.3390/pharmaceutics14081686)
Supplement: Supplementary file 1 [file pharmaceutics-14-01686-s001.zip › pharmaceutics-1839171-supplementary.pdf]

pharmaceutics

MDPI

Supplementary Materials: Zinc(II), Palladium(II), and Metal-Free Phthalocyanines Bearing Nipagin-Functionalized Substituents against *Candida auris* and Selected Multidrug-Resistant Microbes

Daniel Ziental, Dariusz T. Mlynarczyk, Emil Kolasinski, Emre Güzel, Jolanta Dlugaszewska, Łukasz Popenda, Stefan Jurga, Tomasz Goslinski and Lukasz Sobotta

1. NMR Data

1.1. NMR Data for 2

Table S1. ^{1}H and ^{13}C NMR data obtained for **2** including key correlations determined from ^{1}H-^{1}H COSY, ^{1}H-^{13}C HSQC and ^{1}H-^{13}C HMBC spectra.

δ_H (ppm)	Multiplicity ($J_{H\text{-}H}$ in Hz)	^{1}H-^{1}H COSY δ_H (ppm)	^{1}H-^{13}C HSQC δ_C (ppm)	^{1}H-^{13}C HMBC δ_C (ppm)			
7.93	s	-	120.0	152.4	127.8		
7.90	m	6.91	132.1	166.5	163.7	132.1	114.9
6.91	m	7.90	114.9	163.7	123.3	114.9	
5.17	t (6.4)	2.54	72.5	152.4	27.2		
4.30	t (6.4)	2.36	68.8	163.7	27.1		
4.18	t (6.7)	1.54	64.9	166.5	31.4	19.9	
2.54	m	5.17 2.36	27.2	72.5	68.8	27.1	
2.36	m	4.30 2.54	27.1	72.5	68.8	27.2	
1.54	m	4.18 1.29	31.4	64.9	19.9	14.3	
1.29	m	1.54 0.82	19.9	64.9	31.4	14.3	
0.82	t (7.4)	1.29	14.3	31.4	19.9		
0.10	s	-	-				

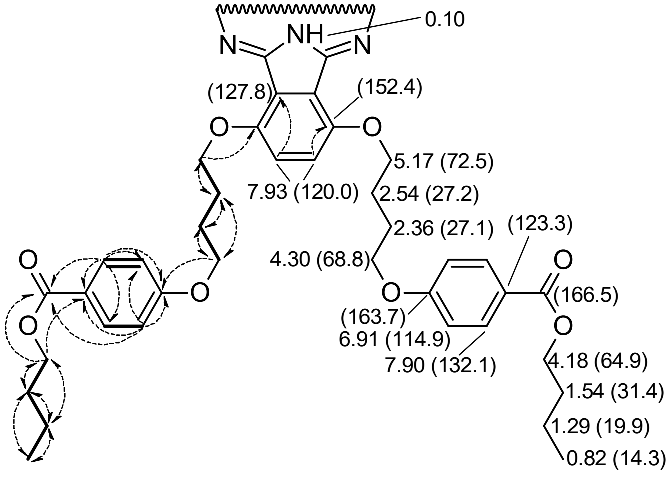

Figure S1. ^{1}H and (^{13}C) chemical shift values [ppm] and key correlations observed in NMR spectra of **2**. Bold lines: ^{1}H-^{1}H COSY; Arrows: ^{1}H-^{13}C HMBC.

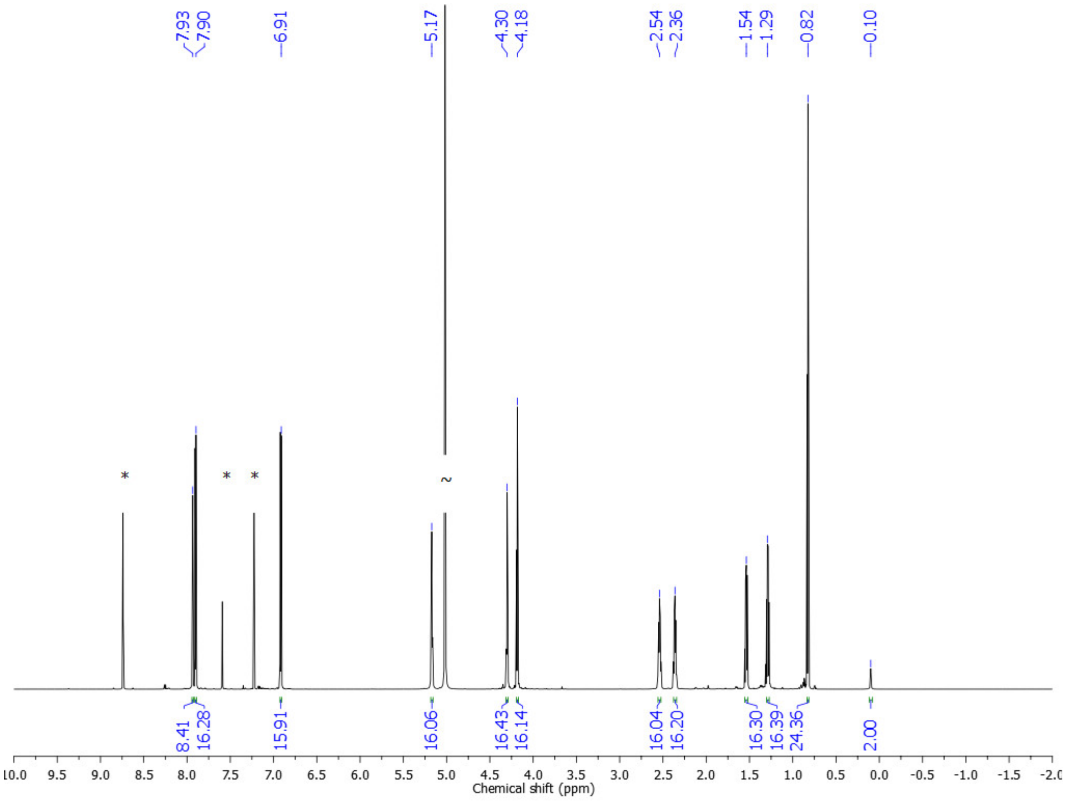

Figure S2. ^{1}H NMR spectrum of **2** (800 MHz, pyridine-*d*5, 298 K). The symbols * and ~ indicate pyridine-*d*5 and water residual peaks, respectively.

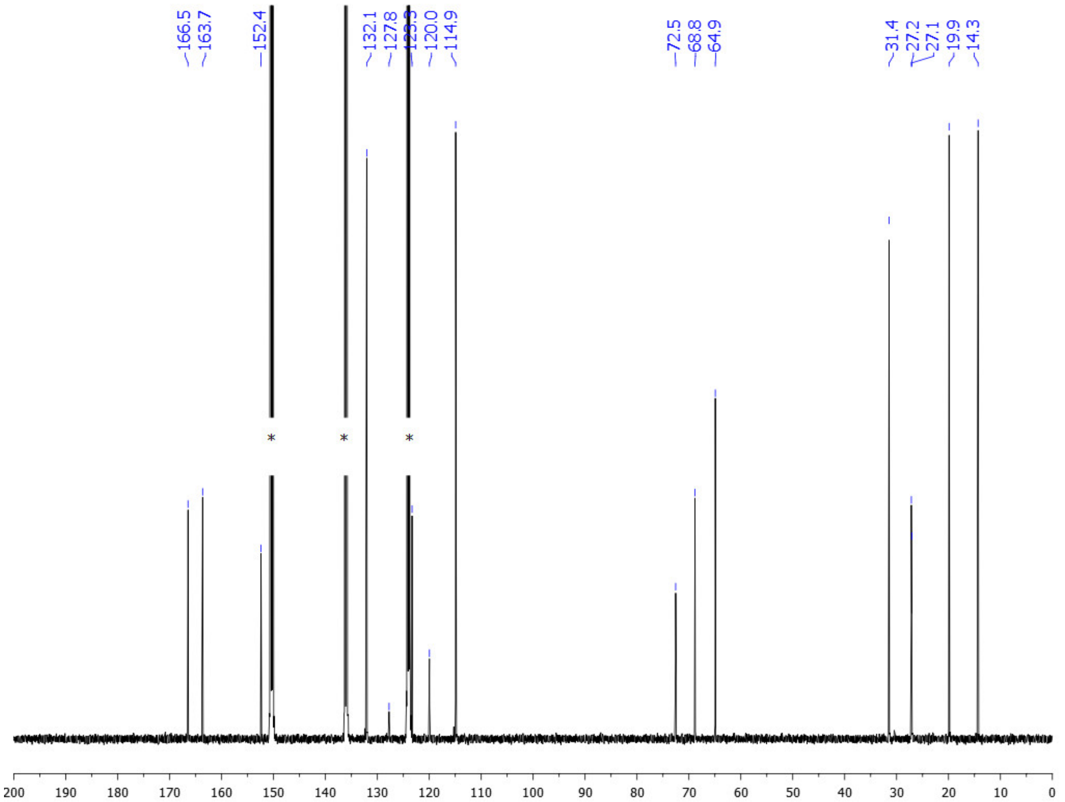

Figure S3. ^{13}C NMR spectrum recorded for 2 (126 MHz, DMSO-*d*6, 298 K). The symbol * indicates DMSO-*d*6 residual peak.

1.2. NMR Data for 3

Table S2. ^{1}H and ^{13}C NMR data obtained for **3** including key correlations determined from ^{1}H-^{1}H COSY, ^{1}H-^{13}C HSQC and ^{1}H-^{13}C HMBC spectra.

δ_H (ppm)	Multiplicity (J_{H-H} in Hz)	^{1}H-^{1}H COSY δ_H (ppm)	^{1}H-^{13}C HSQC δ_C (ppm)	^{1}H-^{13}C HMBC δ_C (ppm)			
8.01	m	6.99	132.2	166.6	163.7	132.2	115.0
7.92	s	-	119.8	153.8	152.5	130.0	
6.99	m	8.01	115.0	163.7	123.5	115.0	
5.26	t (6.5)	2.54	72.9	152.5	27.2		
4.28	2 × t (6.6)	2.33 1.59	68.9 65.0	166.6	163.7	31.5	27.2
				19.9			
2.54	m	5.26 2.33	27.21	72.9	68.9	27.2	
2.33	m	4.28 2.54	27.17	72.9	68.9	27.2	
1.59	m (6.7)	4.28 1.33	31.5	65.0	19.9	14.3	
1.33	m	1.59 0.85	19.9	65.0	31.5	14.3	
0.85	t (7.4)	1.33	14.3	31.5	19.9		

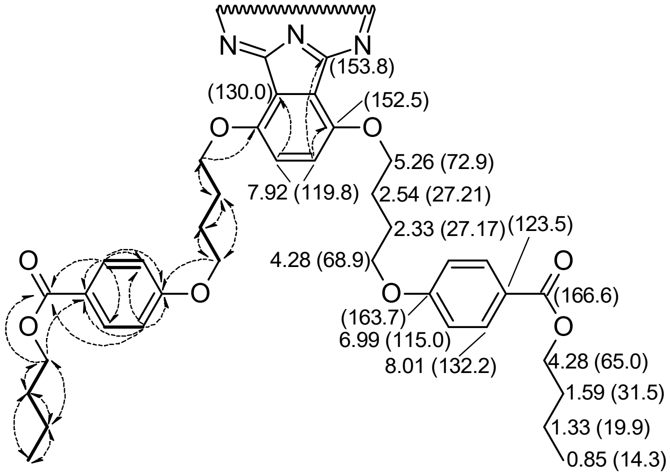

Figure S4. ^{1}H and (^{13}C) chemical shift values [ppm] and key correlations observed in NMR spectra of **3**. Bold lines: ^{1}H-^{1}H COSY; Arrows: ^{1}H-^{13}C HMBC.

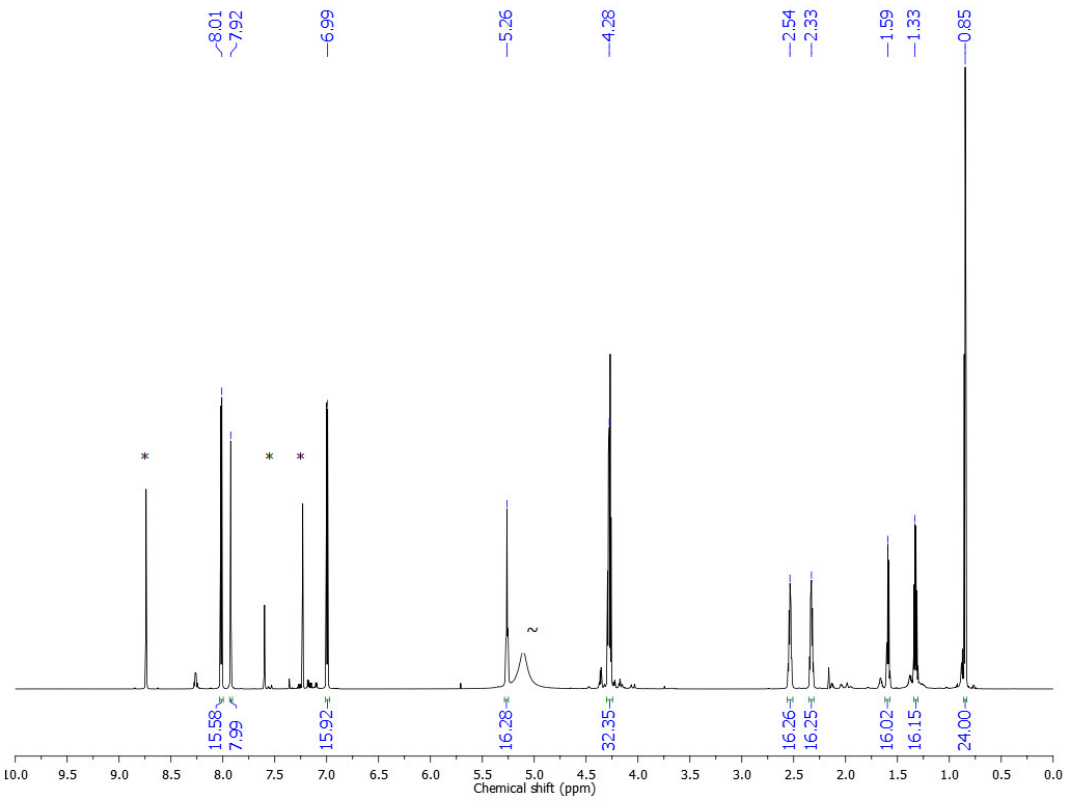

Figure S5. ^{1}H NMR spectrum of **3** (800 MHz, pyridine-*d*5, 298 K). The symbols * and ~ indicate pyridine-*d*5 and water residual peaks, respectively.

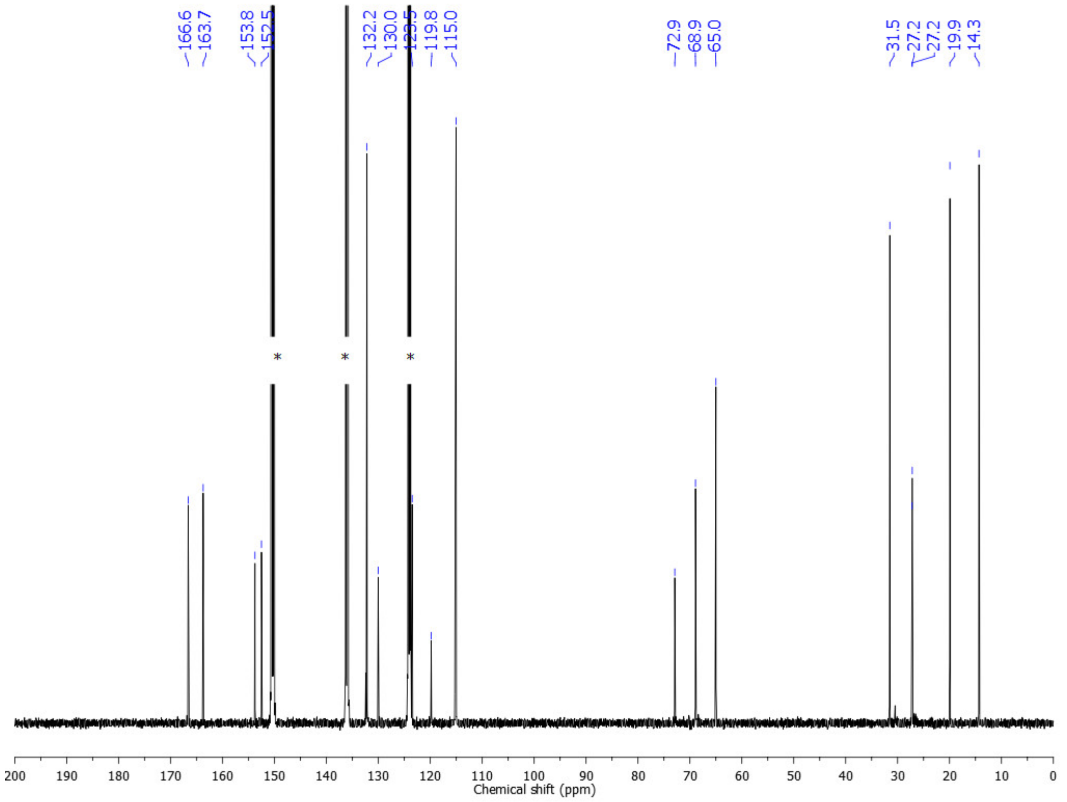

Figure S6. ^{13}C NMR spectrum recorded for 3 (126 MHz, pyridine-d5, 298 K). The symbol * indicates pyridine-d5 residual peak.

1.3. NMR Data for 4

Table S3. ^{1}H and ^{13}C NMR data obtained for **4** including key correlations determined from ^{1}H-^{1}H COSY, ^{1}H-^{13}C HSQC and ^{1}H-^{13}C HMBC spectra.

**δ$_H$ (ppm)**	**Multiplicity ($J_{H\text{-}H}$ in Hz)**	**^{1}H-^{1}H COSY δ$_H$ (ppm)**	**^{1}H-^{13}C HSQC δ$_C$ (ppm)**	**^{1}H-^{13}C HMBC δ$_C$ (ppm)**			
7.93	s	-	119.8	152.1	142.9	128.4	
7.81	m	6.86	131.8	166.2	163.5	131.8	114.7
6.86	m	7.81	114.7	166.2<br>114.7	163.5	131.8	123.1
5.23	t (6.4)	2.54	72.6	152.1	27.1		
4.31	t (6.4)	2.35	68.6	163.5	26.8		
4.11	t (6.7)	1.48	64.7	166.2	31.3	19.8	
2.54	m	5.23, 2.35	26.8	72.6	68.6	27.1	
2.35	m	4.31, 2.54	27.1	72.6	68.6	26.8	
1.48	m	4.11, 1.26	31.3	64.7	19.8	14.2	
1.26	m	1.48, 0.80	19.8	64.7	31.3	14.2	
0.80	t (7.4)	1.26	14.2	31.3	19.8		
Other carbon atoms: 150.6 ppm							

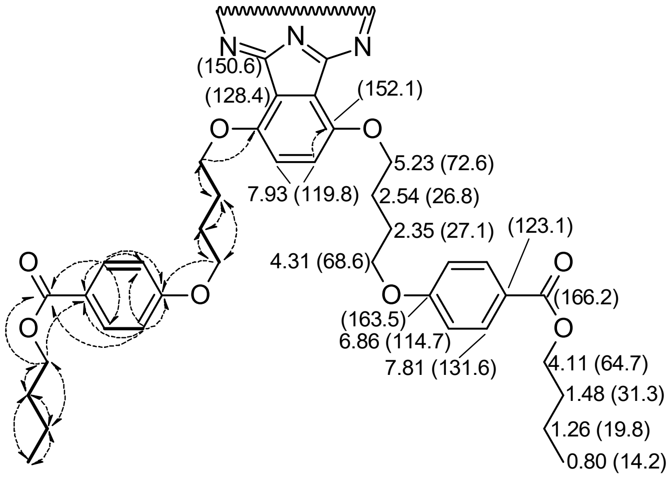

Figure S7. ^{1}H and (^{13}C) chemical shift values [ppm] and key correlations observed in NMR spectra of **4**. Bold lines: ^{1}H-^{1}H COSY; Arrows: ^{1}H-^{13}C HMBC.

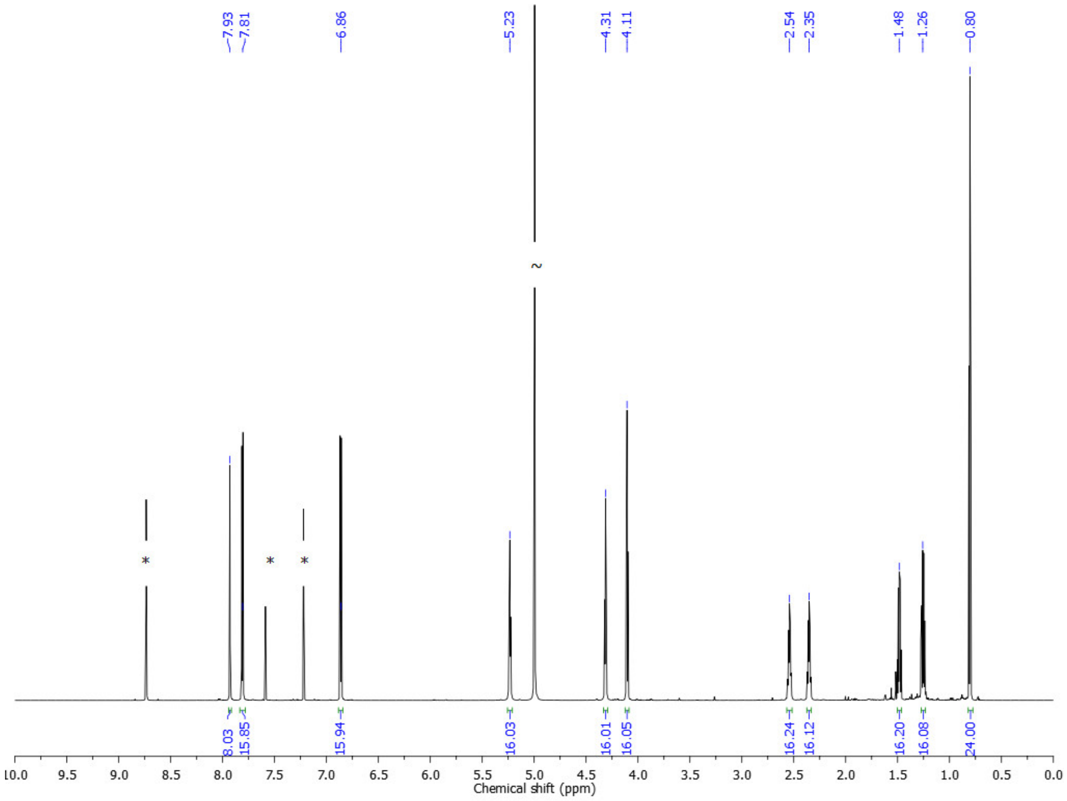

Figure S8. ^{1}H NMR spectrum of **4** (800 MHz, pyridine-d_5, 298 K). The symbols * and ~ indicate pyridine-d_5 and water residual peaks, respectively.

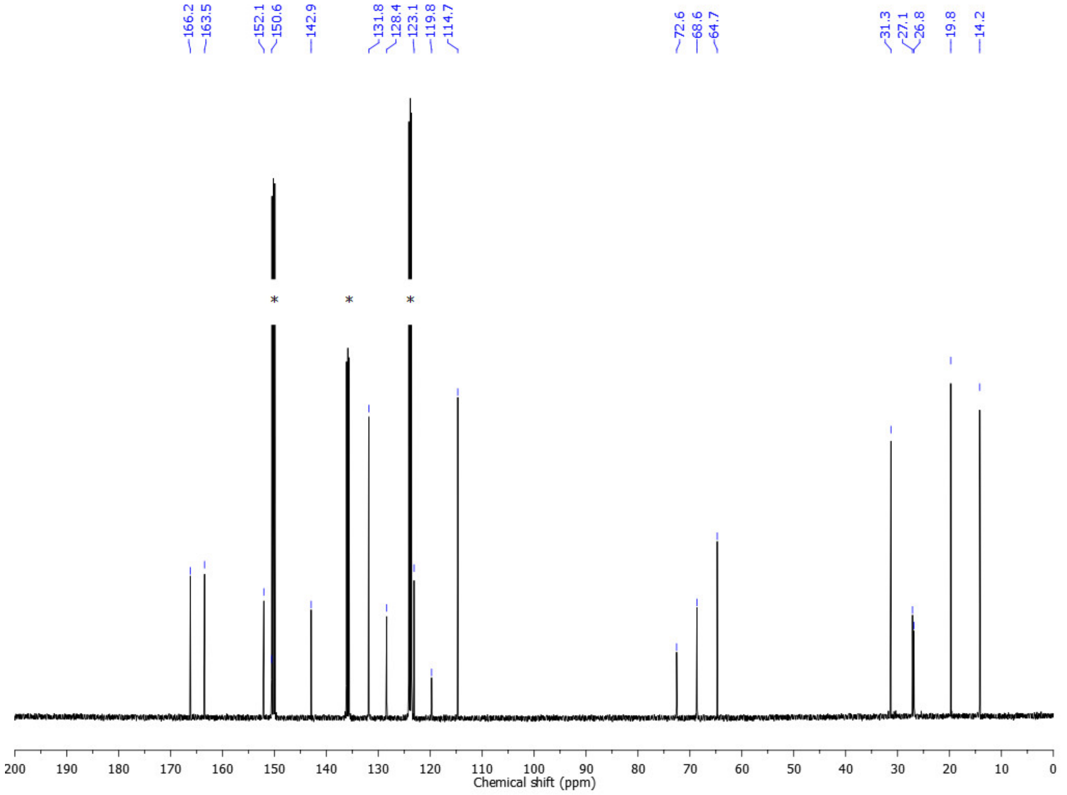

Figure S9. ^{13}C NMR spectrum recorded for **4** (126 MHz, pyridine-d_5, 298 K). The symbol * indicates pyridine-d_5 residual peak.

2. HPLC Analysis

Analytical HPLC was carried out on an Agilent 1200 instrument equipped with a DAD detector. The chromatographic separation was achieved on octadecylsilane coated column, 150 mm × 4.6 mm, 5 µm (Eclipse XDB-C18, Agilent, Santa Clara, CA, USA) using a linear gradient conditions at a flow rate of 1.0 mL/min in different configurations. Separations was performed at 25 °C.

2.1. HPLC of 2

Phases Configuration 1

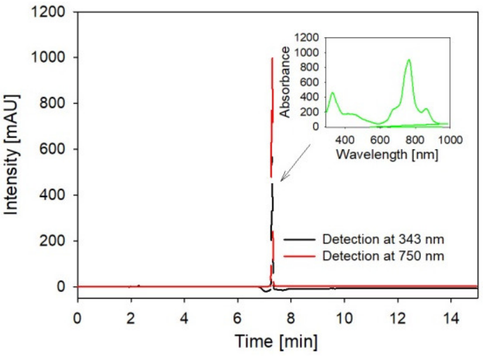

Figure S10. Chromatogram of 2 in phases configuration 1.

Table S4. Separation conditions and parameters of obtained signals for 2 in phases configuration 1.

Mobile phase

Time [min]	ACN [%]	CH_2Cl_2 [%]
0	100	0
4	100	0
5	0	100
15	0	100

Flow	1.0 mL/min
Temperature	25 °C
Column	Agilent, Eclipse XDB-C18 150 mm × 4.6 mm, 5 µm

Detection at λ = 343 nm

Signal	Retention time [min]	Area	Content [%]
1	1.85	10.392	0.71
2	1.98	17.29	1.18
3	2.29	23.91	1.63
4	7.29	1410.96	96.48

Detection at λ = 750 nm

Signal	Retention time [min]	Area	Content [%]
1	7.29	3591.269	100.00

Phases Configuration 2

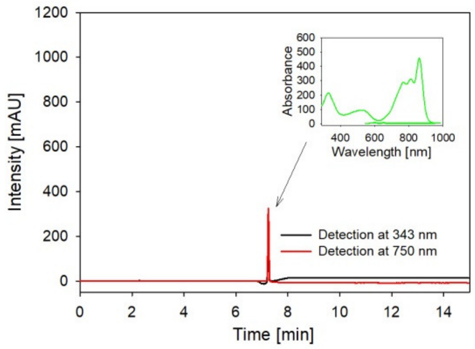

Figure S11. Chromatogram of 2 in phases configuration 2.

Table S5. Separation conditions and parameters of obtained signals for 2 in phases configuration 2.

Mobile phase

Time [min]	ACN [%]	CH_2Cl_2 [%]	THF [%]
0	100	0	0
4	100	0	0
5	0	50	50
15	0	50	50

Flow	1.0 mL/min
Temperature	25 °C
Column	Agilent, Eclipse XDB-C18 150 mm × 4.6 mm, 5 µm

Detection at λ = 343 nm

Signal	Retention time [min]	Area	Content [%]
1	1.98	7.91	0.94
2	2.29	10.98	1.31
3	7.25	819.03	97.75

Detection at λ = 750 nm

Signal	Retention time [min]	Area	Content [%]
1	7.250	983.10	100.00

Phases Configuration 3

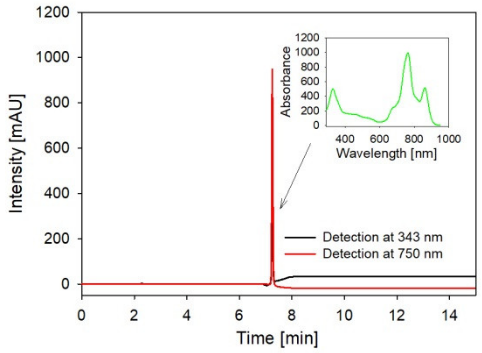

Figure S12. Chromatogram of 2 in phases configuration 3.

Table S6. Separation conditions and parameters of obtained signals for 2 in phases configuration 3.

Mobile phase

Time [min]	ACN [%]	THF [%]
0	100	0
4	100	0
5	0	100
15	0	100

Flow	1.0 mL/min
Temperature	25 °C
Column	Agilent, Eclipse XDB-C18 150 mm × 4.6 mm, 5 µm

Detection at λ = 343 nm				Detection at λ = 750 nm			
Signal	Retention time [min]	Area	Content [%]	Signal	Retention time [min]	Area	Content [%]
1	7.16	394.51	100.00	1	7.25	2948.9	100.00

2.2. HPLC of 3

Phases Configuration 1

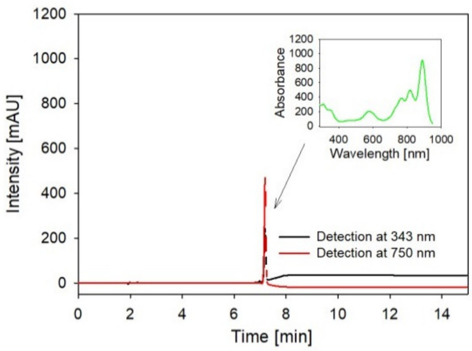

Figure S13. Chromatogram of 3 in phases configuration 1.

Table S7. Separation conditions and parameters of obtained signals for 3 in phases configuration 1.

Mobile phase

Time [min]	ACN [%]	THF [%]
0	100	0
4	100	0
5	0	100
15	0	100

Flow	1.0 mL/min
Temperature	25 °C
Column	Agilent, Eclipse XDB-C18 150 mm × 4.6 mm, 5 µm

Detection at λ = 343 nm				Detection at λ = 750 nm			
Signal	Retention time [min]	Area	Content [%]	Signal	Retention time [min]	Area	Content [%]
1	2.28	29.26	2.69	1	7.191	2023.80	100.00
2	7.19	1059.68	97.31				

Phases Configuration 2

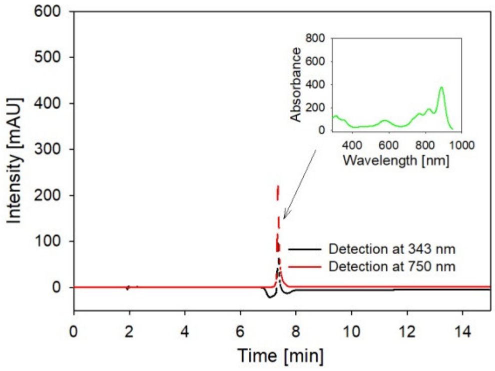

Figure S14. Chromatogram of 3 in phases configuration 2.

Table S8. Separation conditions and parameters of obtained signals for 3 in phases configuration 2.

Mobile phase

Time [min]	ACN [%]	CH_2Cl_2 [%]
0	100	0
4	100	0
5	0	100
15	0	100

Flow	1.0 mL/min
Temperature	25 °C
Column	Agilent, Eclipse XDB-C18 150 mm × 4.6 mm, 5 µm

Detection at λ = 343 nm				Detection at λ = 750 nm			
Signal	Retention time [min]	Area	Content [%]	Signal	Retention time [min]	Area	Content [%]
1	2.28	30.58	4.19	1	7.35	1236.00	100.00
2	7.35	698.46	96.81				

Phases Configuration 3

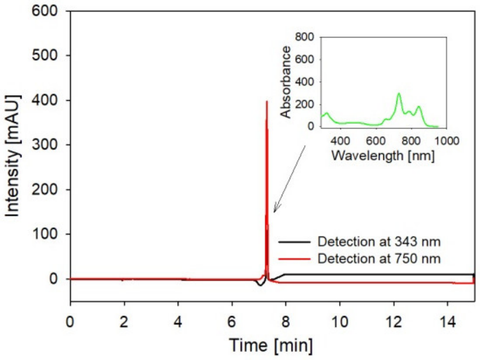

Figure S15. Chromatogram of 3 in phases configuration 3.

Table S9. Separation conditions and parameters of obtained signals for 3 in phases configuration 3.

Mobile phase

Time [min]	ACN [%]	THF [%]	CH_2Cl_2 [%]
0	100	0	0
4	100	0	0
5	0	50	50
15	0	50	50

Flow	1.0 mL/min
Temperature	25 °C
Column	Agilent, Eclipse XDB-C18 150 mm × 4.6 mm, 5 µm

Detection at λ = 343 nm

Signal	Retention time [min]	Area	Content [%]
1	2.28	29.20	3.72
2	7.21	755.46	96.27

Detection at λ = 750 nm

Signal	Retention time [min]	Area	Content [%]
1	7.21	1672.66	100.00

2.3. HPLC of 4

Phases Configuration 1

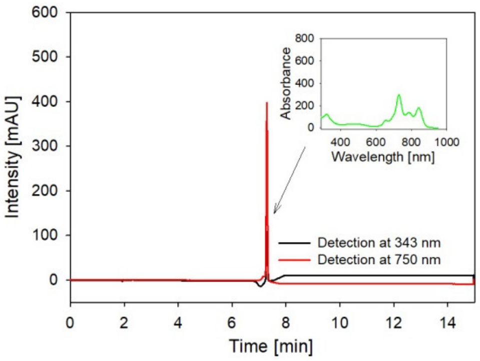

Figure S16. Chromatogram of 4 in phases configuration 1.

Table S10. Separation conditions and parameters of obtained signals for 4 in phases configuration 1.

Mobile phase

Time [min]	ACN [%]	CH_2Cl_2 [%]	THF [%]
0	100	0	0
4	100	0	0
5	0	100	50
15	0	100	50

Flow	1.0 mL/min
Temperature	25 °C
Column	Agilent, Eclipse XDB-C18 150 mm × 4.6 mm, 5 µm

Detection at λ = 346 nm

Signal	Retention time [min]	Area	Content [%]
1	2.31	6.16	1.36
2	7.29	447.23	98.64

Detection at λ = 750 nm

Signal	Retention time [min]	Area	Content [%]
1	7.22	135.63	100.00

Phases Configuration 2

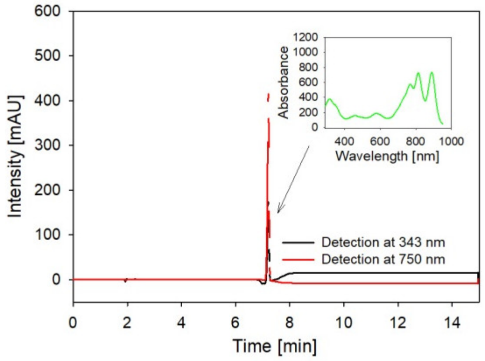

Figure S17. Chromatogram of 4 in phases configuration 2.

Table S11. Separation conditions and parameters of obtained signals for 4 in phases configuration 2.

Mobile phase

Time [min]	ACN [%]	THF [%]
0	100	0
4	100	0
5	0	100
15	0	100

Flow	1.0 mL/min
Temperature	25 °C
Column	Agilent, Eclipse XDB-C18 150 mm × 4.6 mm, 5 µm

Detection at λ = 346 nm

Signal	Retention time [min]	Area	Content [%]
1	1.87	10.39	1.34
2	1.98	12.01	1.55
3	2.29	6.09	0.79
4	7.28	745.40	96,32

Detection at λ = 750 nm

Signal	Retention time [min]	Area	Content [%]
1	7.28	1552.91	100.00

Phases Configuration 3

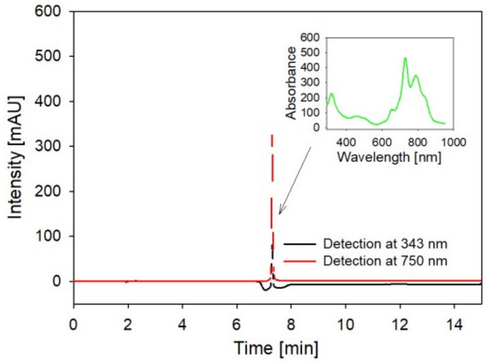

Figure S18. Chromatogram of 4 in phases configuration 3.

Table S12. Separation conditions and parameters of obtained signals for 4 in phases configuration 3.

Mobile phase

Time [min]	ACN [%]	CH_2Cl_2 [%]
0	100	0
4	100	0
5	0	100
15	0	100

Flow	1.0 mL/min
Temperature	25 °C
Column	Agilent, Eclipse XDB-C18<br>150 mm × 4.6 mm, 5 µm

Detection at λ = 346 nm

Signal	Retention time [min]	Area	Content [%]
1	2.29	13.14	3.29
2	7.30	386.72	96.71

Detection at λ = 750 nm

Signal	Retention time [min]	Area	Content [%]
1	7.30	1093.25	100.00

3. High Preasure Xenon Lamp Emission Profile

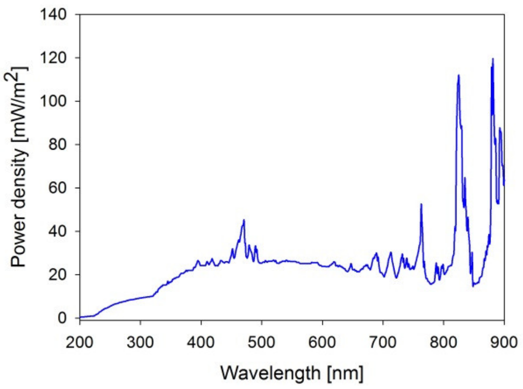

Figure S19. High preasure xenon lamp emission.

4. Dark Toxicity

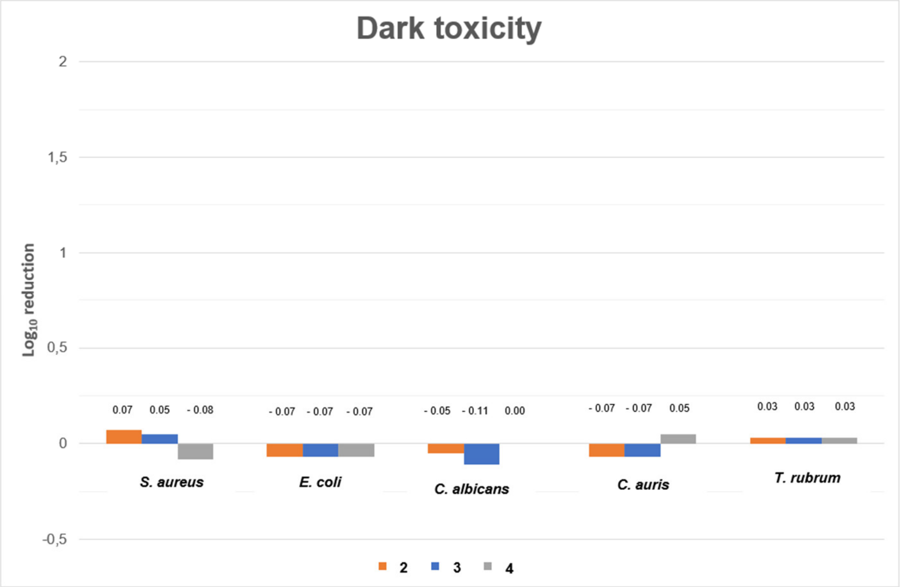

Figure S20. Dark toxicity of studied compounds against bacteria and fungi.
